# Supplementary figures and images for: Zmiz1 is a novel regulator of lymphatic endothelial cell gene expression and function
Source: PLoS One. 2024 May 8;19(5):e0302926. doi: 10.1371/journal.pone.0302926 (PMC11078365; doi:10.1371/journal.pone.0302926)

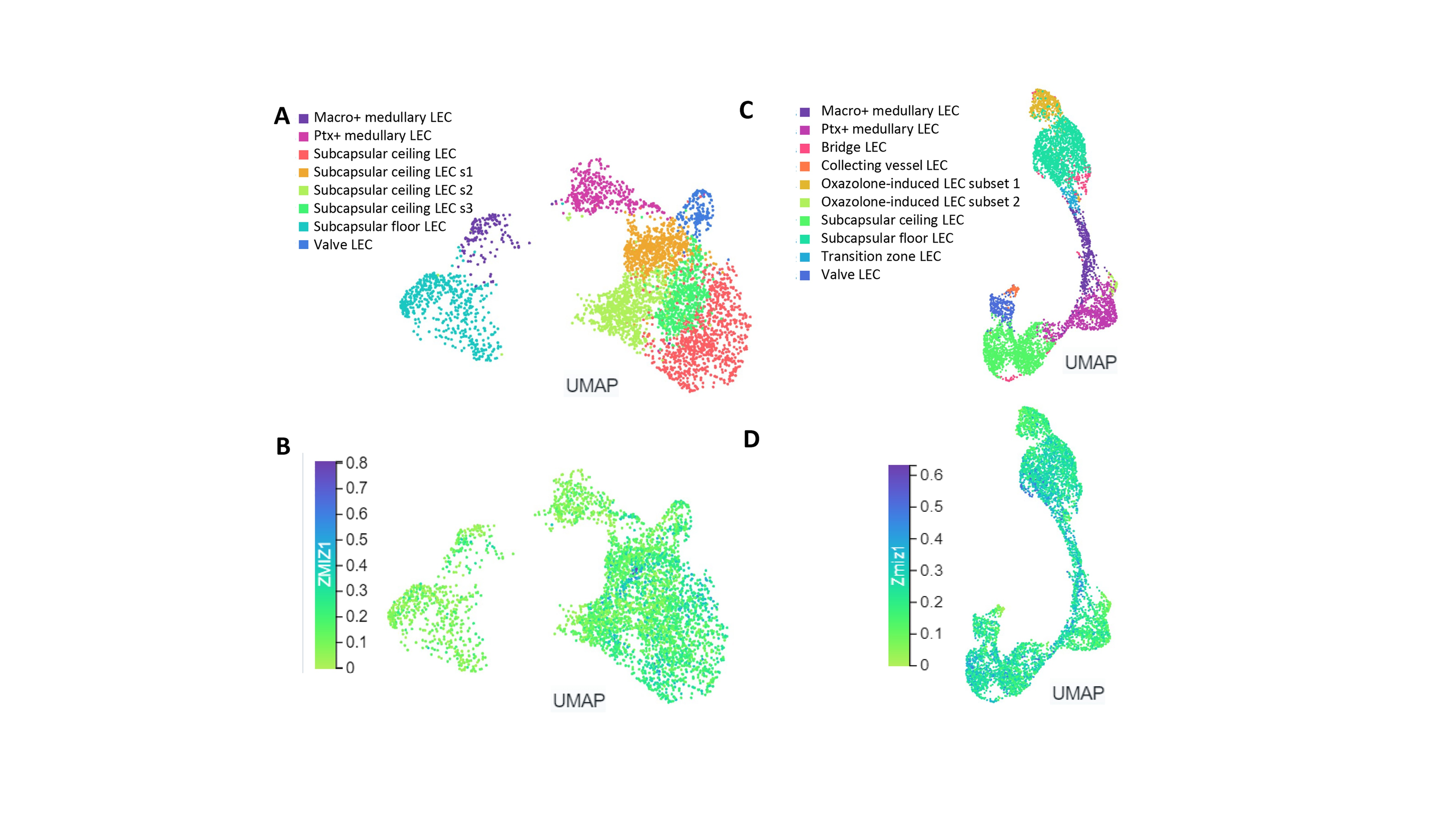

Supplement: S1 Fig — (A, C) Single cell UMAP of human (A) and mouse (C) lymph node lymphatic endothelial cells (LEC) subtypes. (B, D) Zmiz1 expression in distinct LEC subtypes in human (B) and mouse (D) lymph node. Adapted from https://cellxgene.cziscience.com/collections/9c8808ce-1138-4dbe-818c-171cff10e650 [29]. (TIF) [file pone.0302926.s001.tif]

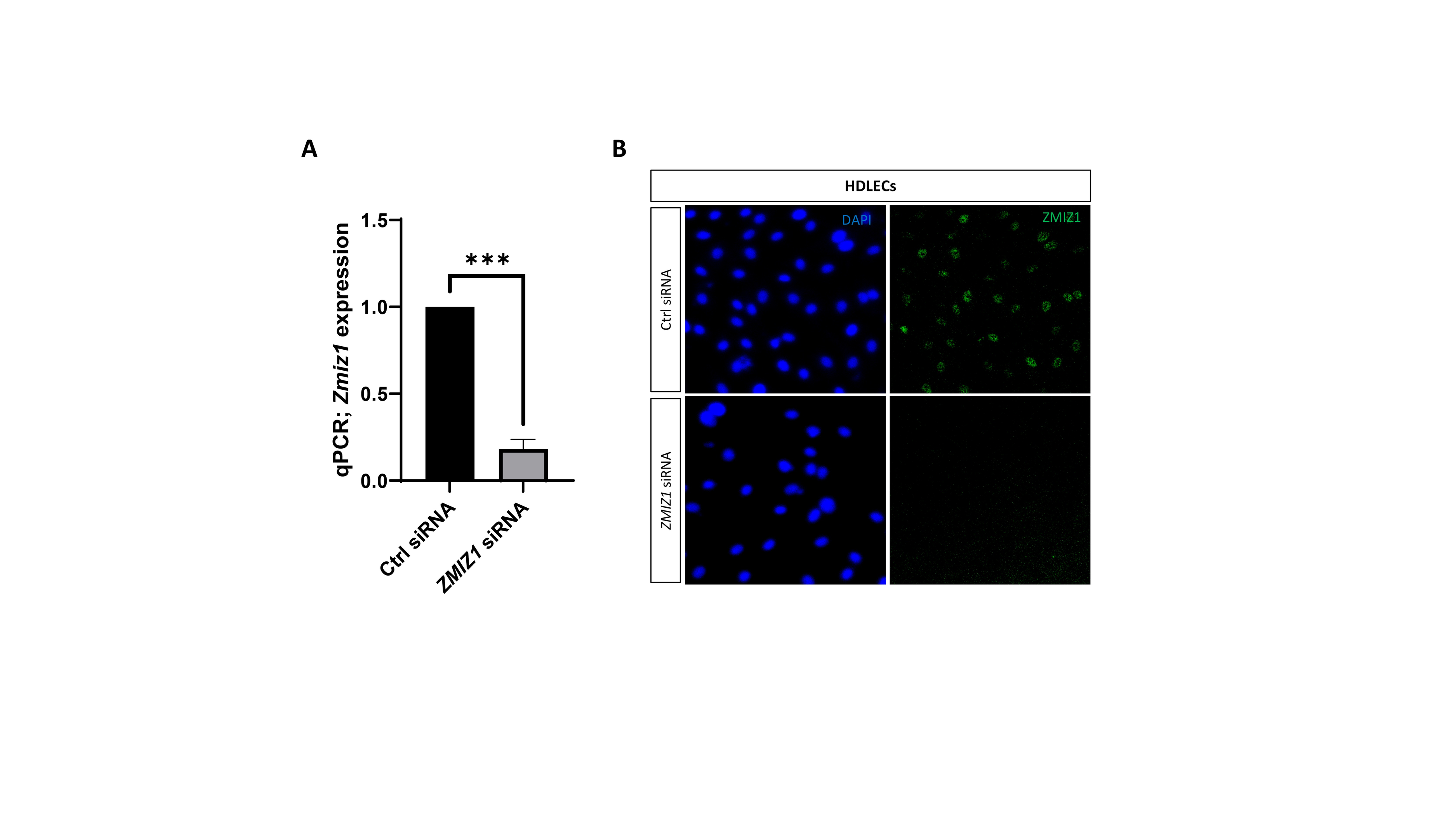

Supplement: S2 Fig — (A) qPCR analysis and (B) immunofluorescent antibody staining for ZMIZ1 confirms loss of Zmiz1 expression in HDLECs treated with ZMIZ1 siRNA, as compared to control siRNA treatments (n = 3). All values are mean ± SEM. ***P < 0.001 calculated by unpaired Student’s t test. (TIF) [file pone.0302926.s002.tif]

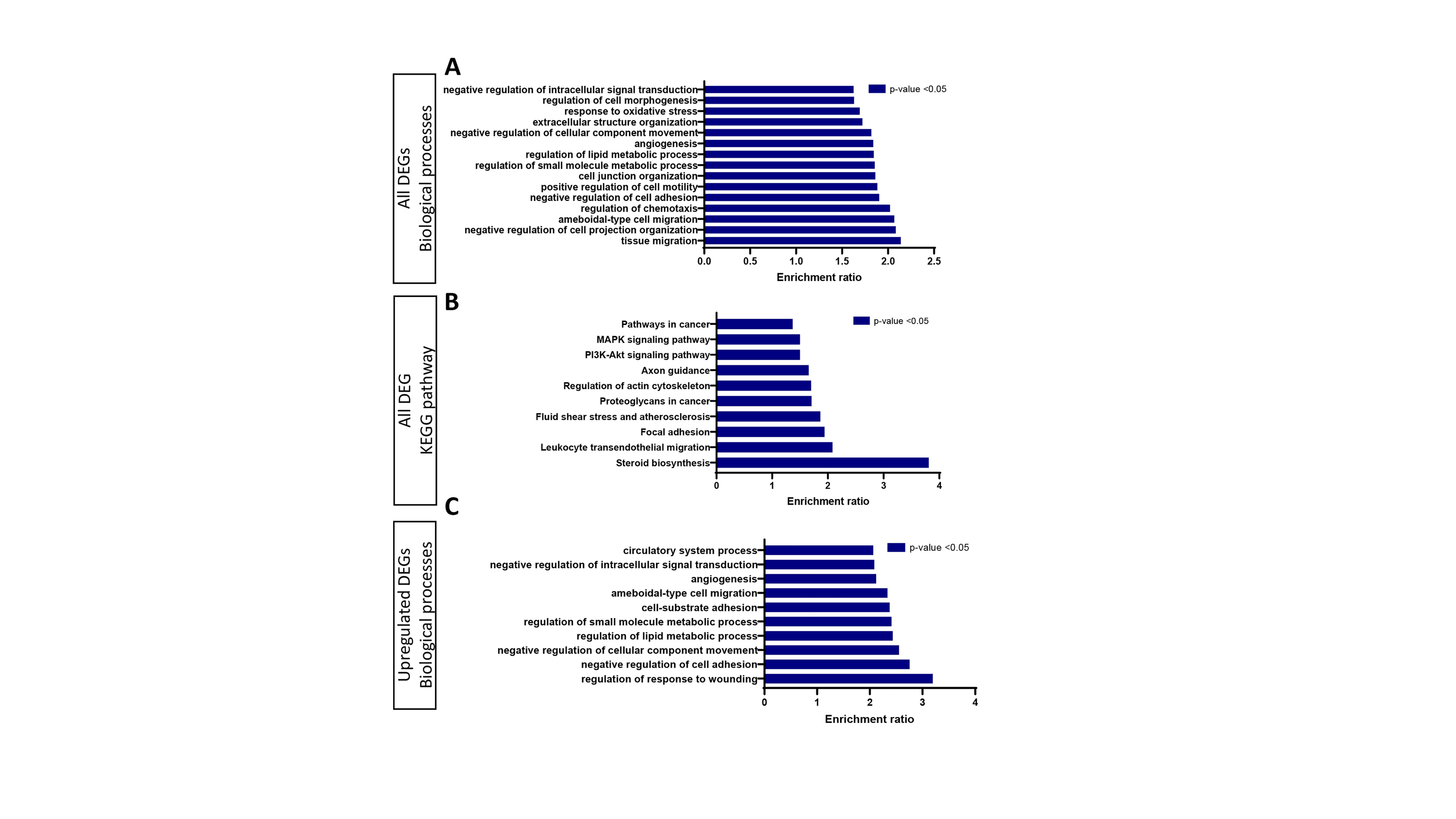

Supplement: S3 Fig — (A-B) Top biological processes (A) and KEGG pathway (B) enriched in both upregulated and downregulated genes following loss of Zmiz1 in HDLECs. (C) Top biological processes enriched in upregulated genes following loss of Zmiz1 in HDLECs. p-value <0.05. (TIF) [file pone.0302926.s003.tif]

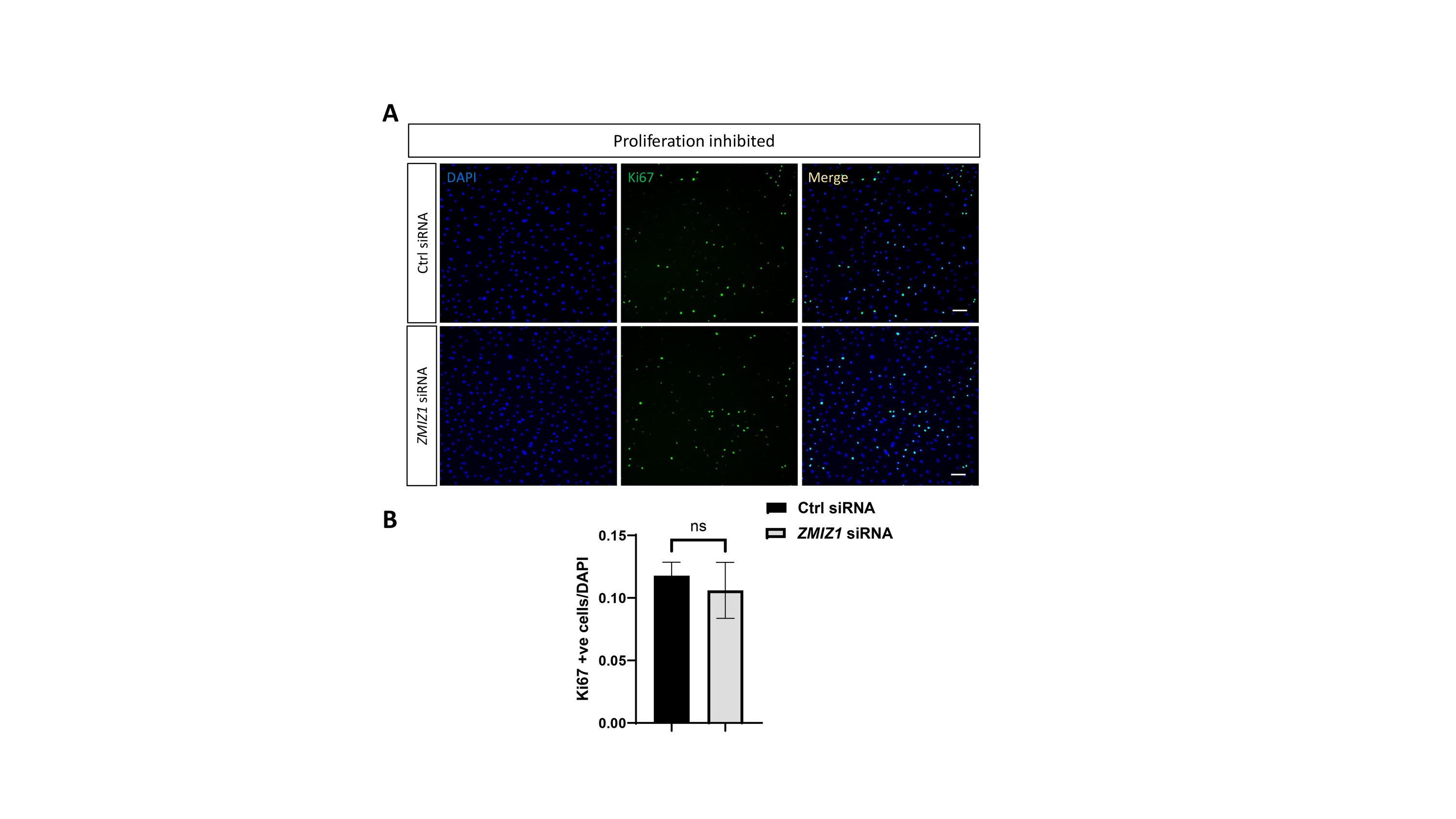

Supplement: S4 Fig — (A) Proliferation inhibition using Cytosine β-D-arabinofuranoside for 3 hours in HDLECs treated with ctrl and ZMIZ1 siRNA. DAPI (blue), Ki67(green). (B) Quantification for Ki67 positive (+ve) cells/DAPI (10X field) show no difference in rate of proliferation (n = 3). All values mean ± SEM. Scale bars: 100 μm. ns–not significant, calculated by unpaired Student’s t test. (TIF) [file pone.0302926.s004.tif]

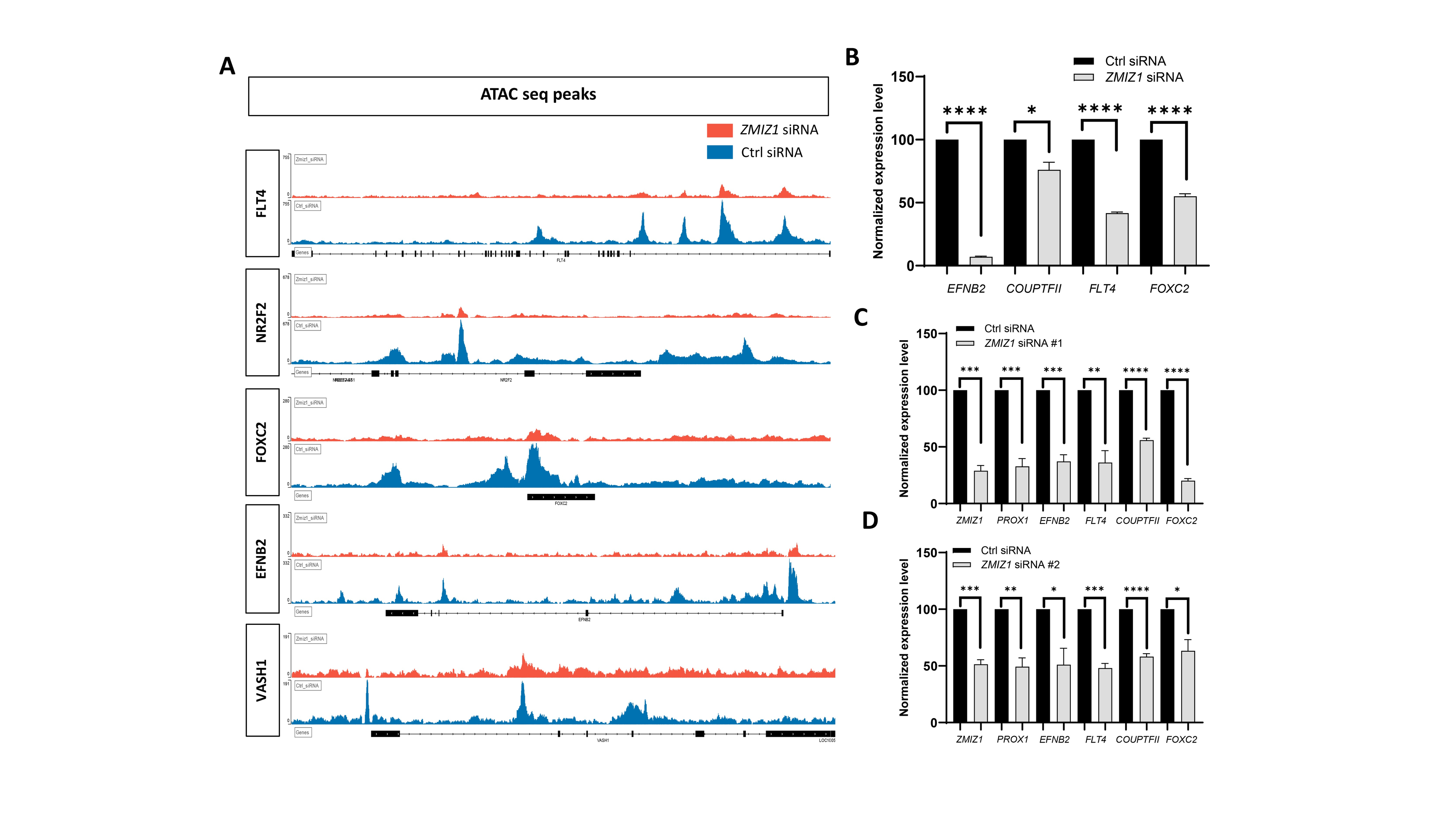

Supplement: S5 Fig — (A) ATAC-seq peaks for FLT4, NR2F2, FOXC2, EFNB2, and VASH1 in control and ZMIZ1 siRNA treated HDLECs. ATAC-seq peaks are colored blue (control (Ctrl) HDLECs) and orange (ZMIZ1 siRNA HDLECs). (B) qPCR analysis of control and ZMIZ1 siRNA treated HDLECs confirm reduced expression of lymphatic development genes EFNB2, COUPTFII, FLT4 and FOXC2 in the absence of Zmiz1 (n = 3). (C-D) qPCR analysis of ZMIZ1, PROX1, EFNB2, FLT4, COUPTFII, and FOXC2 using two different individual Zmiz1 siRNAs from B (n = 3). All values are mean ± SEM. *P <0.05, **P < 0.01, ***P < 0.001, ****P < 0.0001 calculated by unpaired Student’s t test. (TIF) [file pone.0302926.s005.tif]

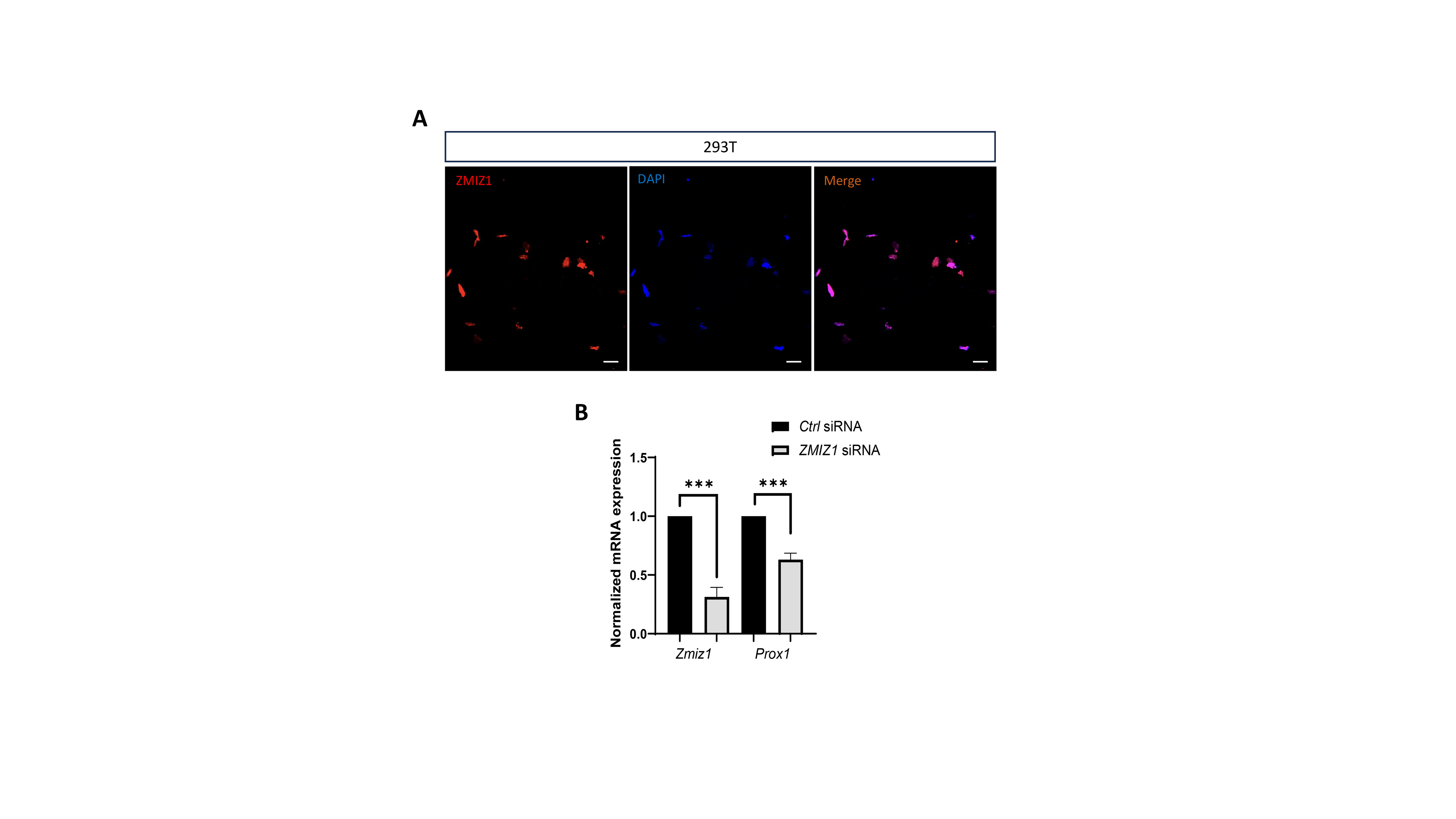

Supplement: S6 Fig — (A) HEK-293T cells fluorescently immunolabeled for ZMIZ1 (red) and DAPI (blue) showed nuclear expression of ZMIZ1. Scale bars: 20 μm (B) qPCR analysis of control and ZMIZ1 siRNA treated HEK-293T cells confirm significant downregulation of both ZMIZ1 and PROX1 mRNAs. n = 3. All values are mean ± SEM. ***P < 0.001 calculated by unpaired Student’s t test. (TIF) [file pone.0302926.s006.tif]

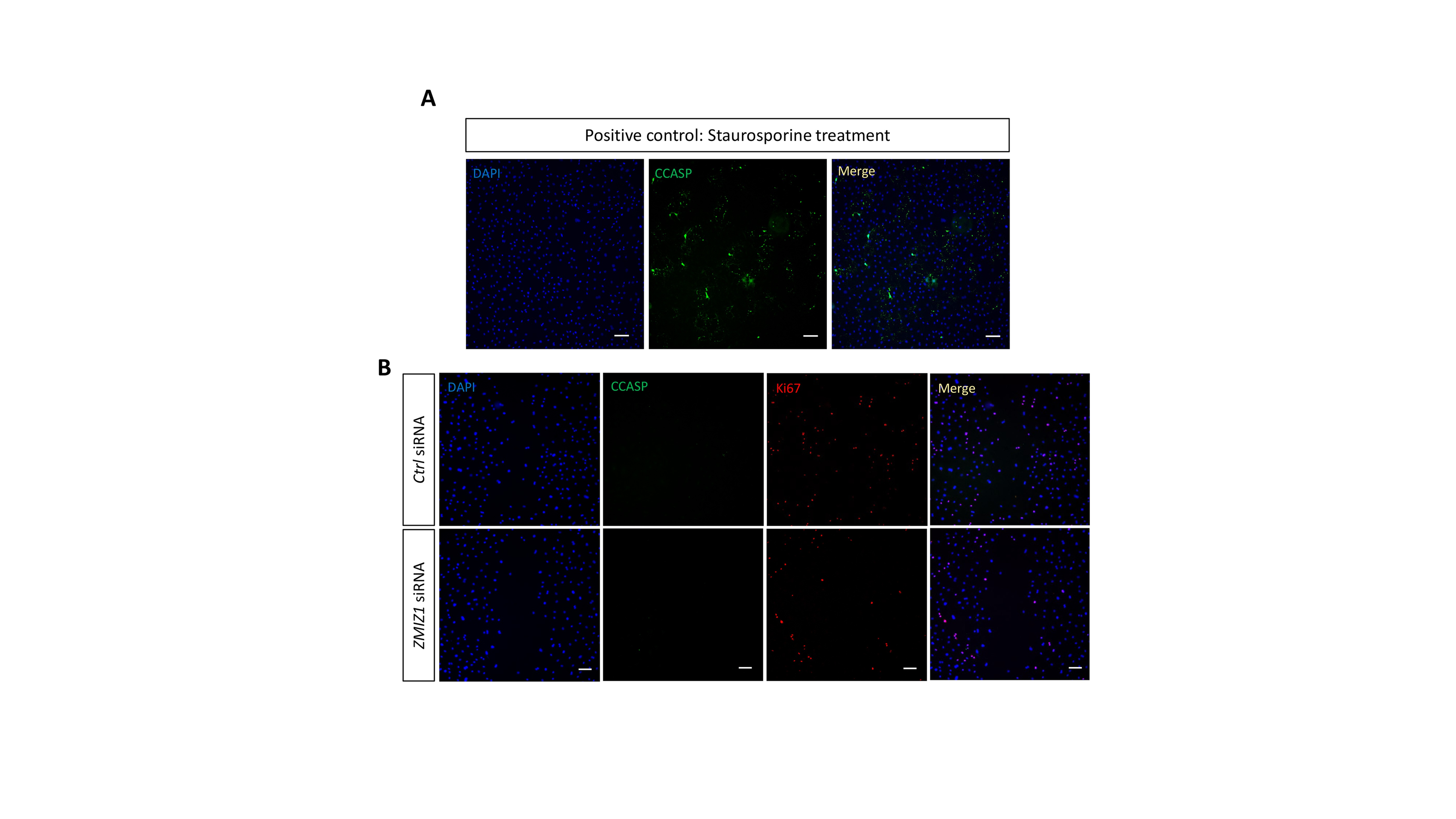

Supplement: S7 Fig — (A) HDLECs treated with Staurosporine, an inducer of cell death, and stained with the apoptotic marker cleaved caspase 3 (CCASP3) serve as a positive control. (B) HDLECS treated with control and ZMIZ1 siRNAs and immunofluorescently labeled for DAPI (blue), CCASP3 (green) and KI67(green). No apoptotic cells were observed in either treatment. n = 3; scale bars: 50 μm. (TIF) [file pone.0302926.s007.tif]

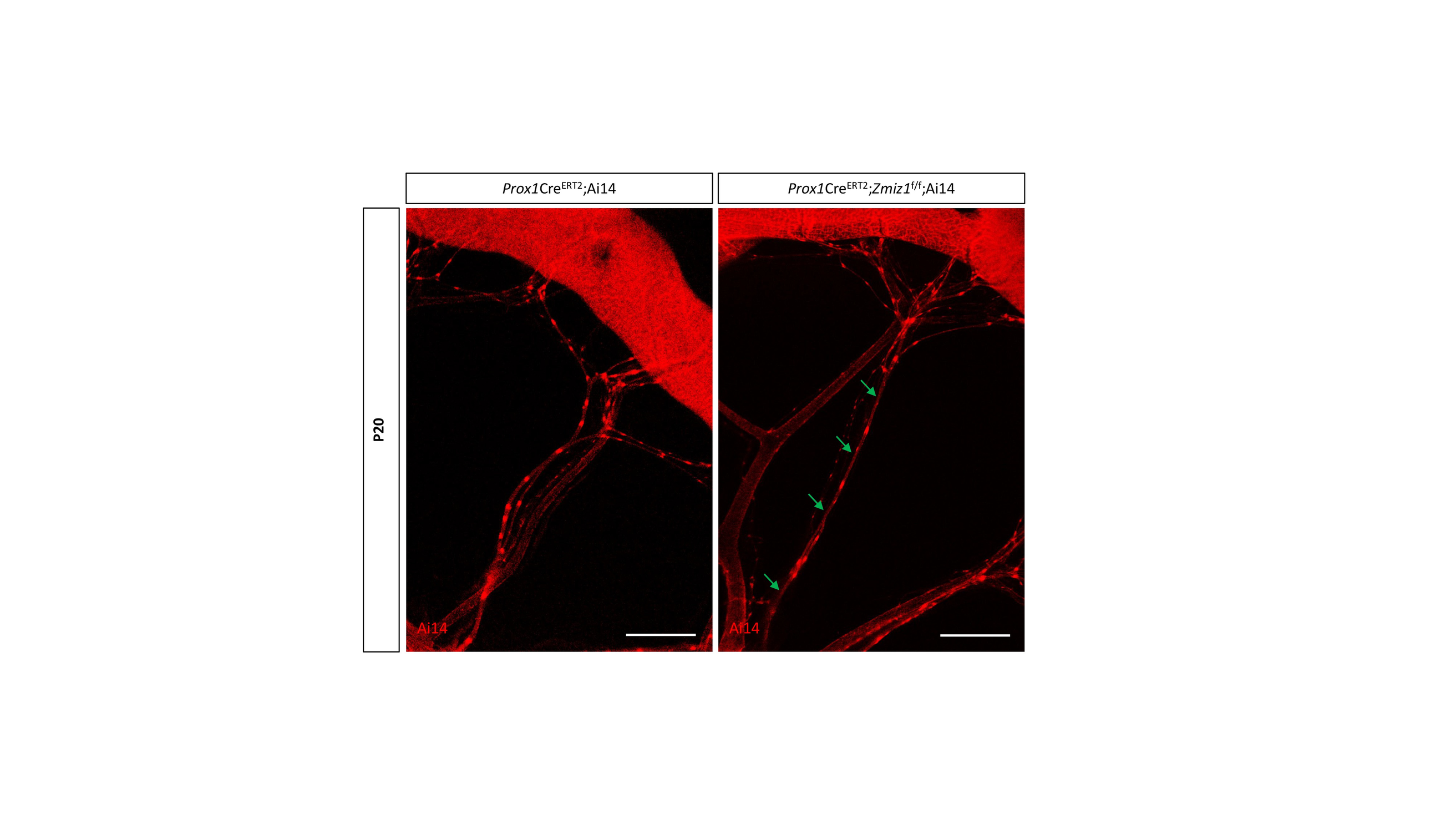

Supplement: S8 Fig — (A) Fluorescence imaging of the morphology of postnatal day (P) 20 mesenteric lymphatic vasculature indicated by RFP expression in Prox1CreERT2;Ai14 control and Prox1CreERT2;Zmiz1f/f;Ai14 mutant mice. Abnormal RFP positive valve arrangements are represented by green arrows. No significant differences in lymphatic vessel diameters were observed between Zmiz1 wild type and mutant mice. Scale bars: 1 mm. Three control and Zmiz1 knockout mesenteries were used. (TIF) [file pone.0302926.s008.tif]

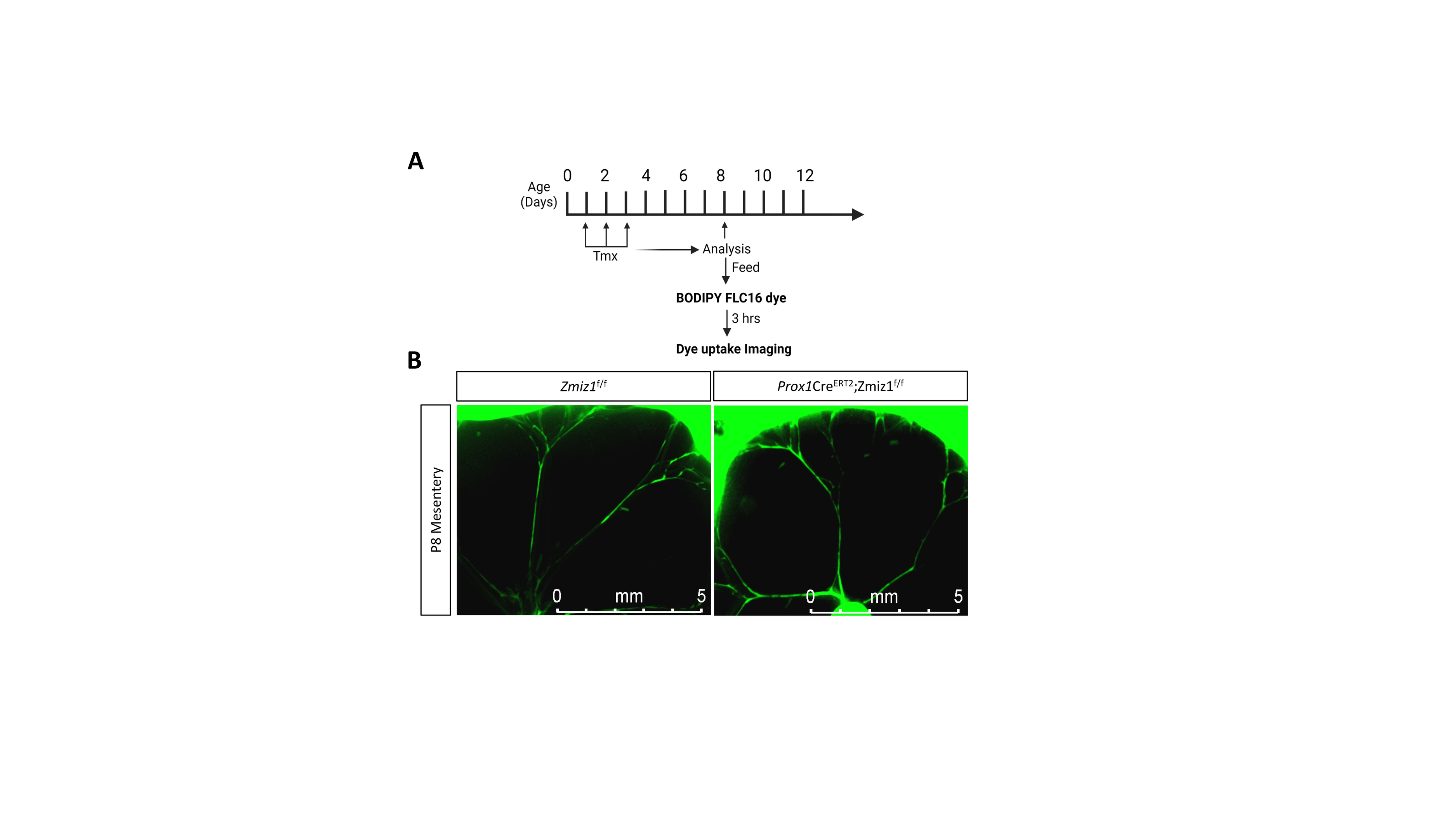

Supplement: S9 Fig — (A) Schematic illustration of postnatal lymph flow test using BODIPY FLC16 dye. (B) Fluorescence images of P8 mesenteric lymphatic vessels in Zmiz1-KO pups indicate that lymph flow is not impaired after the deletion of Zmiz1, as BODIPY FLC16 dye was similarly present throughout the mesenteric lymphatic vessels of control mice (n = 3–5). Scale bars: 5 mm. (TIF) [file pone.0302926.s009.tif]

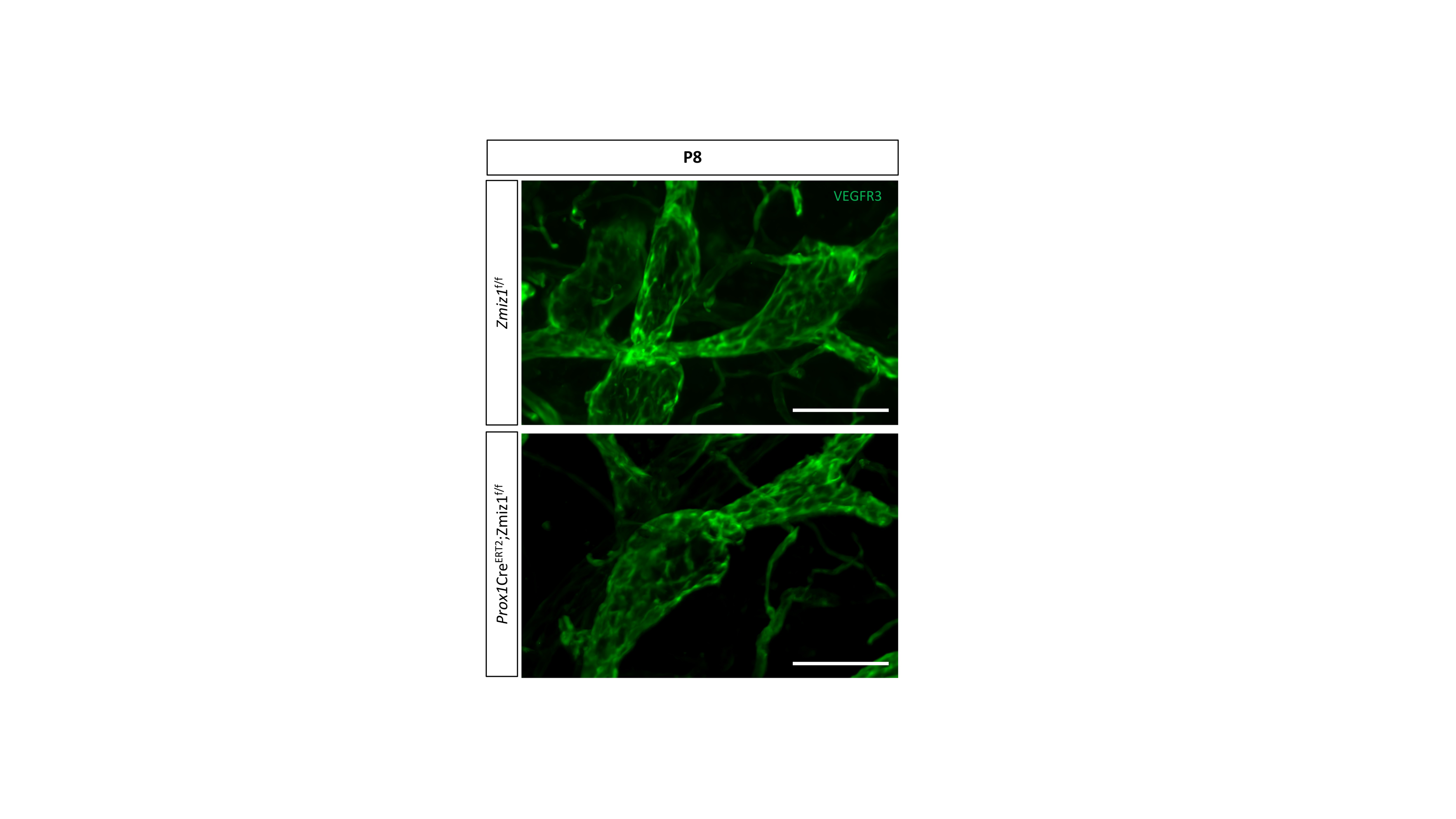

Supplement: S10 Fig — (A) Wholemount immunostaining of VEGFR3 (green) in Zmiz1 wild type and mutant postnatal day (P) 8 ears. No obvious differences in the lymphatic organization, density and valves were noted. Scale bars: 1000 μm. Three control and knockout mice each were examined. (TIF) [file pone.0302926.s010.tif]

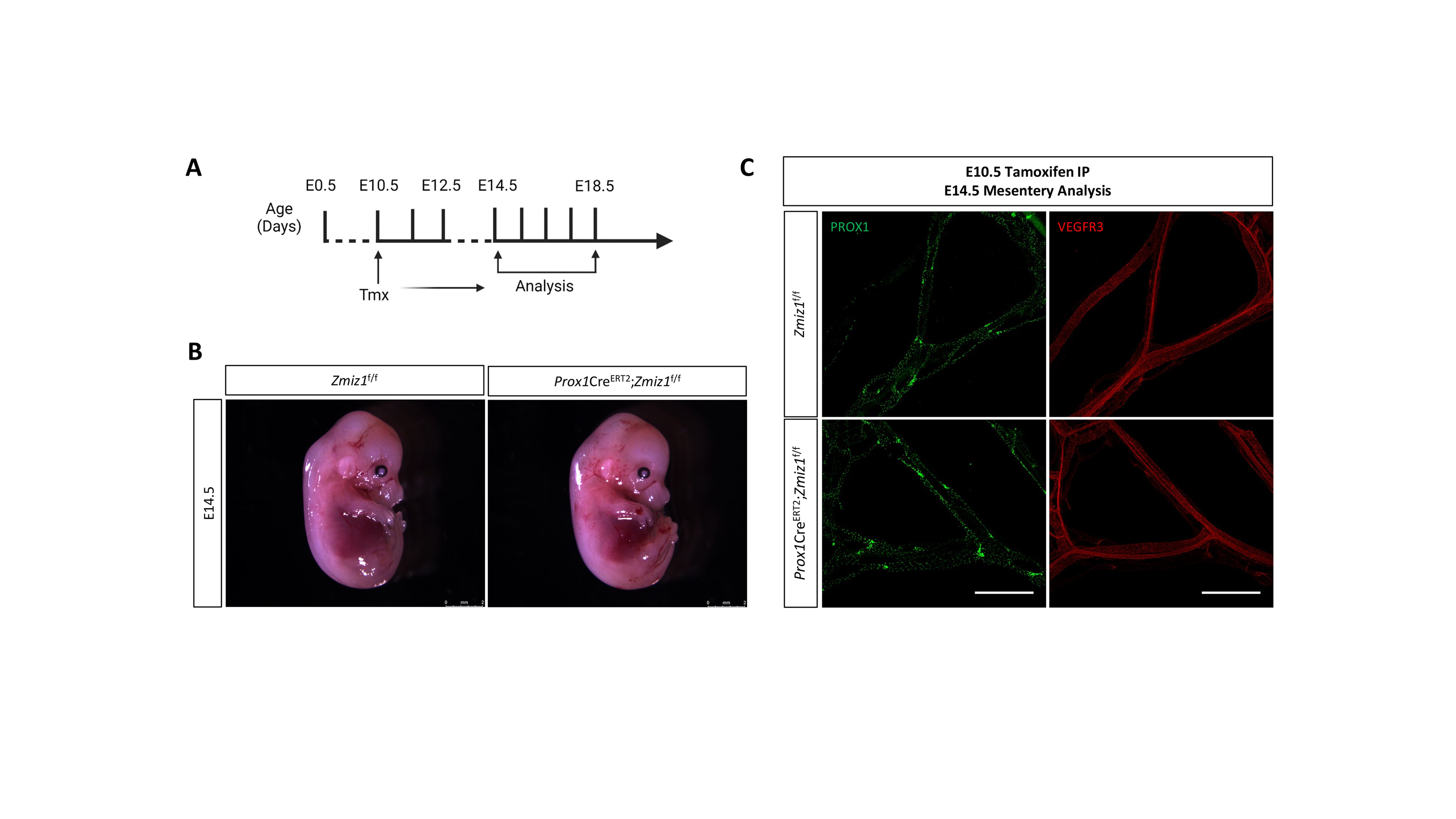

Supplement: S11 Fig — (A) Tamoxifen schedule used for embryonic deletion of Zmiz1. Tmx, tamoxifen. (B) E14.5 control and Zmiz1-KO embryos. No edema was observed at E14.5 (n = 3–5; scale bars: 2 mm). (C) E14.5 control and Zmiz1-KO mesentery immunolabeled for PROX1 (green) and VEGFR3 (red). No differences in PROX1 and VEGFR3 expression or lymphatic vessel morphology were noted (n = 3; scale bars: 1 mm). (TIF) [file pone.0302926.s011.tif]

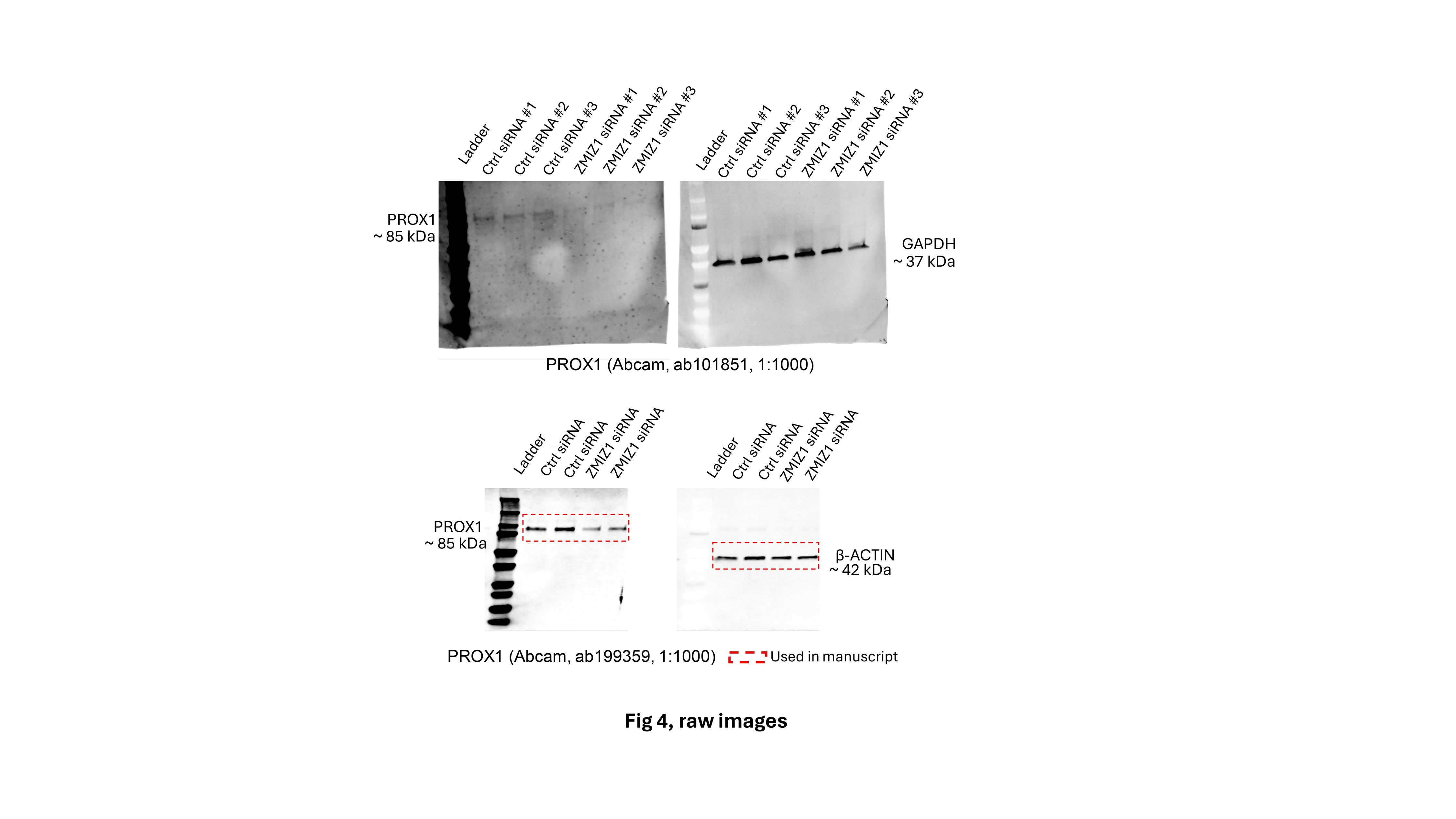

Supplement: S12 Fig — (TIF) [file pone.0302926.s012.tif]

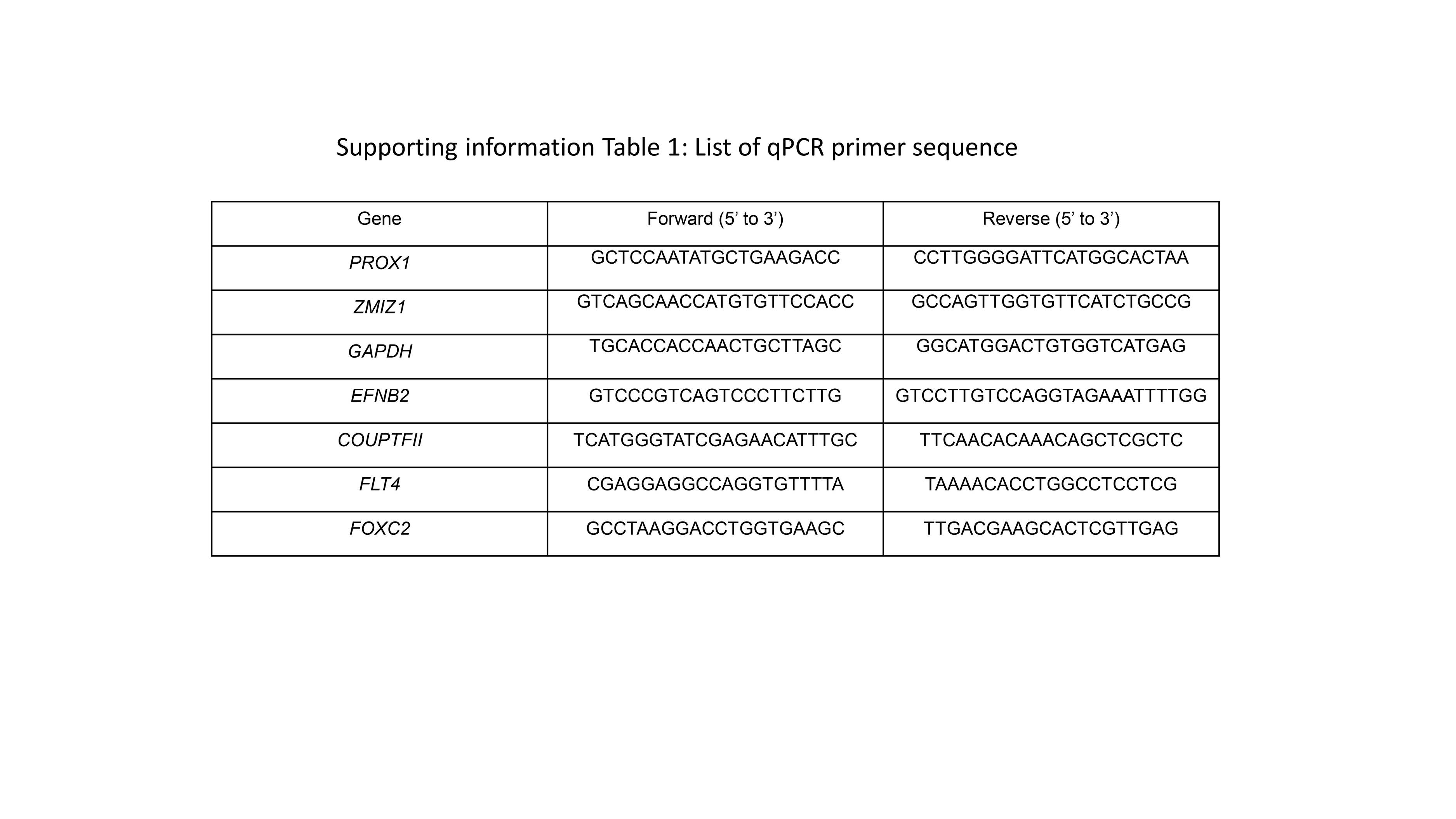

Supplement: S1 Table — (TIF) [file pone.0302926.s013.tif]
