# Supplementary material for: Zmiz1 is a novel regulator of lymphatic endothelial cell gene expression and function
Source: PLoS One. 2024 May 8;19(5):e0302926. doi: 10.1371/journal.pone.0302926 (PMC11078365; doi:10.1371/journal.pone.0302926)
Supplement: Attachment — Submitted filename: Response to reviewers.docx [file pone.0302926.s014.docx]

Response to reviewers

1. Is the manuscript technically sound, and do the data support the conclusions?

The manuscript must describe a technically sound piece of scientific research with data that supports the conclusions. Experiments must have been conducted rigorously, with appropriate controls, replication, and sample sizes. The conclusions must be drawn appropriately based on the data presented.

Reviewer #1: Yes

Reviewer #2: Partly

We appreciate the reviewers’ comments. While our manuscript was in review, we felt we could improve the rigor of the experiments related to the ATAC-seq represented in Fig 3. In the original submission, we identified two open areas of chromatin in the Prox1 gene of HDLECs treated with control siRNA (Fig 3H). One region was previously identified as an enhancer for Prox1 expression. However, upon Zmiz1 siRNA treatment, the accessibility of the open chromatin at these two sites was markedly reduced suggesting that loss of Zmiz1 was responsible for the changes in open chromatin status. Therefore, to further assess Zmiz1 regulation of Prox1 at these sites, we conducted luciferase reporter assays. We cloned the Prox1 peaks identified from the ATAC-seq experiments into luciferase reporter vectors and performed the assay in HEK-293T cells. We note that lymphatic ECs, including HDLECs, were not used because they are notoriously difficult to transfect and HEK-293T cells are routinely used as an alternative. We observed that upon Zmiz1 siRNA treatment, luciferase activity was significantly reduced in comparison to control siRNA treated cells indicating that Zmiz1 regulates Prox1 expression at these two identified regions of DNA. Subsequently, we decided to include this experiment into the manuscript because we felt it further supported the ATAC-seq results and overall findings of the study.

2. Has the statistical analysis been performed appropriately and rigorously?

Reviewer #1: Yes

Reviewer #2: Yes

No comment

3. Have the authors made all data underlying the findings in their manuscript fully available?

The PLOS Data policy requires authors to make all data underlying the findings described in their manuscript fully available without restriction, with rare exception (please refer to the Data Availability Statement in the manuscript PDF file). The data should be provided as part of the manuscript or its supporting information, or deposited to a public repository. For example, in addition to summary statistics, the data points behind means, medians and variance measures should be available. If there are restrictions on publicly sharing data—e.g. participant privacy or use of data from a third party—those must be specified.

Reviewer #1: Yes

Reviewer #2: Yes

All data incorporated in this manuscript is fully available. As mentioned in the manuscript, RNA-seq and ATAC-seq data have been deposited in the Gene Expression Omnibus (GEO) database with accession no. GSE225130 and GSE225057 respectively.

4. Is the manuscript presented in an intelligible fashion and written in standard English?

PLOS ONE does not copyedit accepted manuscripts, so the language in submitted articles must be clear, correct, and unambiguous. Any typographical or grammatical errors should be corrected at revision, so please note any specific errors here.

Reviewer #1: Yes

Reviewer #2: Yes

No comment
