# Supplementary material for: Zmiz1 is a novel regulator of lymphatic endothelial cell gene expression and function
Source: PLoS One. 2024 May 8;19(5):e0302926. doi: 10.1371/journal.pone.0302926 (PMC11078365; doi:10.1371/journal.pone.0302926)
Supplement: Attachment — Submitted filename: Response to reviewers_FINAL.docx [file pone.0302926.s015.docx]

**Additional data added but not requested by the reviewers:**

In reviewing our own manuscript, we felt we could improve the rigor of the experiments related to the ATAC-seq represented in Fig 3. In the original submission, we identified two open areas of chromatin in the Prox1 gene of HDLECs treated with control siRNA (Fig 3H)—one region was previously identified as an enhancer for Prox1 expression. However, upon Zmiz1 siRNA treatment, the accessibility of the open chromatin at these two sites was markedly reduced suggesting that loss of Zmiz1 was responsible for the changes in open chromatin status. Therefore, to further assess Zmiz1 regulation of Prox1 at these sites, we conducted luciferase reporter assays. We cloned the two Prox1 peaks identified from the ATAC-seq experiments into luciferase reporter vectors and performed the assays in HEK-293T cells. We note that lymphatic ECs, including HDLECs, were not used because they are notoriously difficult to transfect and HEK-293T cells are routinely used as an alternative. We observed that upon Zmiz1 siRNA treatment, luciferase activity was significantly reduced in comparison to control siRNA treated cells further supporting that Zmiz1 regulates Prox1 expression at these two identified regions of DNA. We have included this experiment into Fig 3I, J of the manuscript because we felt it further supported the ATAC-seq results and overall findings of the study. In addition, and as Supplemental Figure 6, we showed that ZMIZ1 is expressed in the nucleus of HEK-293T cells via immunostaining and that the ZMIZ1-siRNA lead to a significant reduction in both *ZMIZ1* and *PROX1* mRNA compared to control siRNA in HEK-293T cells.

**Reviewer1:**

Zmiz1 is a novel regulator of lymphatic endothelial cell gene expression and function

This manuscript by Rajan et al; about a novel transcriptional regulator of Prox-1 is interesting. The data is sound, and experiments were planned in a logical fashion. Data presentation and manuscript are well written and easy to follow. Invitro expts. were conducted thoroughly and results were inferred nicely. Despite a strong down regulation of Prox-1 and other lymphatic genes it is surprising that the inducible TG mice didn’t show any major lymphatic phenotype except decreased valve density. I have couple of suggestions in this regard as mentioned below.

1. It is surprising why the authors didn’t pursue any expts. with adult mice? What is the lymphatic structure and function in adult mice in cre activated Zmiz1 mice? What is the status of valves in skin?

We completely understand the reviewer’s point of view regarding experiments associated with adult mice. We were also surprised to find relatively minor lymphatic defects in the neonate Zmiz1-KO mice (addressed in more detail further down) and felt adult studies would be interesting and important to understanding the overall role of Zmiz1 in lymphatic blood vessel structure and function. With that said, we stuck to in vitro and transcriptomic methodologies, and in vivo assessment in early neonate mice because my lab is familiar with these approaches and stages, and because our experience with the lymphatic system is limited. In truth, this work is our introduction into the lymphatic vascular field and we seldom perform any experiments using adult mice. To carry out many of the adult experiments suggested by the reviewer would require a more trial and error approach and expansion of tool sets, which would be difficult to achieve in the given revision time frame. By no means are we trying to minimize the validity of the reviewer’s comments and suggestions, as we also think they are important. We are merely trying to be practical in the self-assessment of our capabilities and time constraint imposed by doing adult studies. Additionally, we feel these suggested experiments wouldn’t necessarily change the overlying implication of the paper that Zmiz1 is a regulator of lymphatic endothelial gene expression, especially Prox1, and note the reviewer reported no issues with the original experiments conducted. We certainly aim to increase our knowledge base and technical skill sets with lymphatic system-based studies going forward, but at the same time would submit that if we had performed adult studies and seen consequences in lymphatic functions, we would have been inclined to send this work to a higher impact journal. Ultimately, we feel adult work could be a separate project to further assess Zmiz1’s role in mesenteric lymphatics, ear skin lymphatics, dorsal skin lymphatics, heart lymphatics, and perhaps by performing more complex and challenging lymphatic valve pressure myography assays. We hope the reviewer understands our point of view and the subsequent responses to their additional comments/suggestions.

In reference to the reviewer’s first suggestions, we did perform an additional analysis aimed at the skin lymphatics in neonate mice. We were able to analyze the lymphatics in the ear skin via VEGFR3 immunostaining of Zmiz1 control and mutant P8 pups. Upon examination, we did not detect any clear changes in lymphatic vessel organization, density, diameters, or valve density. This result indicated that Zmiz1 is not required for lymphatic development in the early postnatal ear skin and has been added to the manuscript text and as Supporting Figure 10. Additionally, and in relationship to the nature of the reviewer’s comments, we analyzed the mesenteric lymphatics of Zmiz1 control and mutant pups at P20. In this case, we observed distinct alterations in valve density and organization, similar to P8 Zmiz1 mutant mice. Though not explicitly addressing the adult angle, we at least provide some data demonstrating that the mesentery lymphatic valve defects persist in slightly older Zmiz1-KO mice. This data has been referenced in the paper and included as Supporting Figure 8. The corresponding images are also shown below:

Regarding the absence of major lymphatic defects despite the downregulation of Prox1 and other major lymphatic players, we offer some possible explanations. Surveying the upregulated genes in Zmiz1-KD HDLECs, we found an enrichment of multiple pro-lymphangiogenic genes (Supporting Figure 3). Genes such as TGFb and Klf4 were among the upregulated genes. Increased expression of these genes, at least in part, may explain the lack of major lymphatic defects. TGF-β signaling maintains the structure of lymphatic vessels and lymphatic homeostasis such that TGF-β KO result in abnormal lymphatic vessel structure, increased lymphatic vessels network, decreased lymphatic drainage, and potentiate lymph angiogenesis (Fukasawa *et al.*, 2021; Itoh and Watabe, 2022). Klf4 is well known for its regulation of angiogenesis. In lymphatics, Klf4 deletion resulted in defective lymphatic vessels, branching morphogenesis and decreased lymphatic density (Choi *et al.*, 2017) and is a valve forming gene (Scallan *et al.*, 2021). Furthermore, valve formation is a critical step during lymphatic vessel development and multiple genes, including PROX1, GATA2, FOXC2, and FOXO1 play important roles. Intriguingly, FOXO1 is significantly downregulated in Zmiz1-KD HDLECs. FOXO1 is a repressor of lymphatic mesenteric valves and FOXO1-KO mice exhibit increased mesenteric lymphatic vessels and valve numbers (Niimi *et al.*, 2021; Scallan *et al.*, 2021). Perhaps increased FOXO1 levels are counterbalancing the lymphatic valve defects induced in Zmiz1-KO mice. These potential explanations have been added to the discussion, which originally included references to i) Zmiz2 compensating in some manner for the loss of Zmiz1; based on our survey of single cell sequencing databases, Zmiz2 is also expressed in the various subtypes of lymphatic ECs and ii) potential differences in the in vivo and in vitro settings.

2. With significantly decreased LEC proliferation and migration may be Zimz1 mice might have some abnormalities in lymphatic structure and function in inflammation, infection wound healing etc. it is worth trying at least one model of adult lymphatic vascular remodeling expt. With these mice.

We appreciate the reviewer’s comment and agree that it would be interesting to know whether loss of Zmiz1 and associated decreases in LEC proliferation and migration leads to changes in lymphatic structure and function in inflammation, infection wound healing, etc. However, we feel these experiments, though worthy and informative, are i) not trivial in nature, especially considering our lack of familiarity with them; ii) are somewhat beyond the scope of our manuscript linking Zmiz1 to transcriptional regulation of lymphatic gene expression; and iii) would be best addressed by future experiments aimed at further characterizing lymphatic structure and function in Zmiz1 deficient mice.

We would also like to note that, for future direction, it is worth looking at the role of Zmiz1 in adulty mice and perform in depth in vivo analysis from looking at lymphatic density, vessel coverage, gut lymphatics, inflammations, wound healing, and infections.

3. Have you maintained these mice beyond P8 and observed their weight gain and lipid absorption after 3-4 wks of age.

We have let a few tamoxifen induced mice go for up to 3 weeks, but we did not observe any noticeable weight gain or other obvious problems typically related to dramatic lymphatic defects. As referenced in #1, we did find that similar to P8, lymphatic valves are present in fewer numbers in Zmiz1-KO mice at P20 (new S8 Fig). However, we did not perform additional BODIPY FL C_16_ tests at 3-4 wks of age because we felt this might not be necessary considering the same valve phenotypes at P8 were also present at P20, and we didn’t see defects in absorption of BODIPY FL C_16_ and subsequent lymphatic flow at P8. In terms of other methodologies to assess lipid absorption, they were not performed but would be interesting to see in future studies.

4. In figure 4 decreased valve density is obvious. I also notice reduced lymphatic diameter in Zmiz1 mice.

We appreciate the reviewer’s keen observations on the differences in lymphatic diameters shown between Zmiz1 control and mutant mesentery in Fig 4. Although the specific Zmiz1-KO mutant shown indicates reduced vessel diameter compared to controls, this was not a consistent phenotype. We initially thought this might be the case, but as we assessed more experimental samples, no significant differences in vessel diameter were observed. Since the images shown could inadvertently suggest a change in diameter to the readers, we made a point in the figure legend to note that differences in diameter were not observed between Zmiz1 control and mutant vessels. We also include images below for the author highlighting the similar vessel diameters between Zmiz1 control and mutant mesentery.

5. I strongly recommend some more in vivo analysis of these mice related to lymphatic density, diameter of skin and gut lymphatics. With such a significant reduction in valve density these mice should have pumping defects in skin lymphatic vessels.

We appreciate Reviewer 1 for commenting on the need for more *in vivo* experiments. As noted in our response to comment #1, we analyzed the ear skin lymphatics at P8 and saw no gross discernable differences in valve morphology or lymphatic vessel density, diameter, and organization (S10 Fig). We also noted in comment #4 that mesenteric lymphatic vessel calibers did not appear significantly different between control and Zmiz1-KO mice (P8 and P20). This is referenced in the Figure 4 legend. For these reasons, we did not provide further in-depth in vivo experiments but cannot rule out the possibility that there are pumping defects in the skin lymphatics or in adults. As mentioned before, we fully believe these types of in-depth in vivo analyses are valid and critical to future directions aimed at further elucidating the role of Zmiz1 in lymphatic structure and function, especially in adults.

**Reviewer2:**

The author investigated the role of Zmiz1 in lymphatic endothelial cells (LECs).

The finding that it is a regulator of the expression of Prox1, which is critically important in LECs, presents interesting data. The finding of the effect on lymphatic valve formation using the knockout mice is of some significance.

However, the presentation of the data is not sufficient, and explanations are lacking in some points.

The following points need to be improved.

Major points: additional experiments are required.

1. Sumoylation of Prox1 controls its ability to induce VEGFR3 expression and lymphatic phenotypes in endothelial cells (J Cell Sci, 2009). The article above suggests that PIAS is involved in SUMOylating Prox1. Can overexpression of Zmiz1 induce SUMOylaton of Prox1 and affect Prox1 function by e.g. Prox1 protein stability or Prox1 nuclear accumulation?

As mentioned by Reviewer 2, studies have shown Prox1 SUMOylation effecting vegfr3 expression. Zmiz1 is a member of PIAS proteins based on structural domains, however PIAS activity of Zmiz1 has not been assessed extensively, and SUMOylation activity of Zmiz1 has only been associated with the androgen receptor (Sharma *et al.*, 2003). In this study, we focused on the *transcriptional role of Zmiz1 in regulating lymphatic endothelial cell gene expression, including Prox1*. The SUMOylation aspect of Zmiz1 is an interesting point, as it is unknown if Zmiz1 might regulate Prox1 function via SUMOylation. However, though interesting and worthy of future investigation, we feel that the potential role of Zmiz1 in Prox1 SUMOylation and its subsequent function go beyond the scope of this study, and therefore, we did not pursue this line of investigation.

2. Although described as ‘similar to other cell types such as Hela,’ since there is no data for HeLa, the author needs to show immunostaining for comparison, along with BEC types such as HUVEC, which would be valuable.

Based upon the nature of the reviewer’s request, instead of solely referencing the study with Hela cells, we now reference proteinatlas.org (Human protein atlas database: <https://www.proteinatlas.org/ENSG00000108175-ZMIZ1/single+cell+type)> and other various publications indicating expression of Zmiz1 in multiple cell types and in the nucleus. In addition, we can confirm that Zmiz1 is expressed in HUVECs, as well as other endothelial cell lines (human teloHAEC and mouse MS1) and in the endothelial cells of mice (see below figure). However, this data is being used for a manuscript in preparation detailing Zmiz1’s role in angiogenesis and therefore we have not included it in this paper. We hope Reviewer 2 understands our reasoning. I have included some of the data in good faith showing that Zmiz1 is expressed in endothelial cells from different databases, as well as in the retinal blood vessels of mice, as revealed in our studies:

**Summary of EC-specific Zmiz1 expression within various tissues. A,** Analysis of *Zmiz1* expression levels in ECs and total tissue of P7 brain, kidney, liver, lung, and adult brain using the Vascular Endothelial Cell Trans-Omics Resource Database (VECTRDB). **B,** *Zmiz1* expression in each cell type isolated from an adult mouse brain can be visualized within the violin plots. **C,** Analysis of *Zmiz1* expression levels in P7 isolated brain ECs and its subtypes using single cell RNA-seq data in VECTRDB. **D,** Representative immunofluorescent images of P7 retinas stained with IB4 and Zmiz1 (A - artery and V - vein). Scale bars: 200 μm. **E,** Expression levels of *Zmiz1* in murine retinal endothelial cells during postnatal development determined using the available bulk RNA sequencing data.

We also note that we tried numerous anti-Zmiz1 antibodies but were never able to detect Zmiz1 in the different endothelial cell lines via western blot or immunofluorescent staining. Rather, we had to use qPCR to show reduced Zmiz1 expression in the cell lines subjected to siRNA targeting Zmiz1. However, and for the reviewer’s reference only, we showed that an HA-tagged version of ZMIZ1 could be detected in the nucleus of mouse MS1 cells:

3. Also, since the specificity of the antibodies was not shown, compare with Zmiz1-knocked down samples.

To address this request, we have performed immunostaining for ZMIZ1 upon Zmiz1-KD in HDLECs. As expected, Zmiz1 antibody is specific to ZMIZ1 since it is undetected in Zmiz1-KD HDLECs. This experiment has been added to Supporting Figure 2B, which now shows loss of Zmiz1 mRNA and protein in Zmiz1-siRNA treated HDLECS. It is shown here for reference:

4. Fig. 1G is quite important data, since the expression of genes with important functions in lymphatic vessels other than Prox1 is shown to be decreased. qRT-PCR at the mRNA level should show the decreased expression. Also, in siRNA experiments, it is necessary to use two or more siRNAs because it is necessary to resolve the suspicion of off-target effects. The author should show using qRT-PCR that the gene changes seen in Fig,1G (Prox1, FLT4, NR2F2, Foxc2, EFNB2, NRP2) are commonly seen as effects of at least two different siRNAs.

We agree that multiple siRNAs are necessary to provide robustness to the study and are critical to have efficient knockdown that does not also exhibit off-target effects. Accordingly, the Zmiz1 siRNA used in our studies consists of 4 individual siRNAs targeting multiple sites of the Zmiz1 transcript. Zmiz1 siRNA were obtained as a SMARTpool ON-TARGETplus siRNA, which is a mixture of 4 siRNA provided as a single reagent; providing advantages in both potency and specificity from horizon discovery at <https://horizondiscovery.com/en/gene-modulation/knockdown/sirna/products/on-targetplus-sirna-reagents>. To clarify this point in the paper, we have noted in the material and methods section that the Zmiz1 siRNA is a collection of 4 siRNAs targeting different regions of the Zmiz1 mRNA. We also note that the same smart pool siRNA for Zmiz1 was used in HUVEC, teloHAECs and MS1 studies that showed efficient knockdown of Zmiz1 expression in those cell lines (different manuscript in preparation).

For the second part referencing expression of other genes important for lymphatic vessel formation, we have used qPCR analysis in HDLECS, as suggested by the reviewer. We confirmed the downregulation of several major lymphatic development genes; Prox1, Flt4, CoupTFII, Foxc2, and Efnb2. Prox1 qRTPCR data is already in Figure 4C in the original manuscript and additional qRT-PCR for Flt4, CoupTFII, Foxc2, and Efnb2 have been added to Supporting Figure 5B, which also include the ATAC-seq peak analysis of several of these noted developmental genes. We felt this data would fit more neatly in this arrangement as opposed to trying to correspond it with the RNA-seq data in Figure 1G.

5. Since cell proliferation is decreased by siRNA, it is not easy to conclude that the results of the wound healing assay are due to decreased cell migration. The author should provide data by using an alternative method.

This potential issue is resolved between Figure 2 and Supporting Figure 4 in the original manuscript where we controlled proliferation using the cell cycle arrest, DNA synthesis inhibitor cytosine β-D-arabinofuranoside. We showed that 1) cytosine β-D-arabinofuranoside treatment leads to similar rates of proliferation in control and Zmiz1 siRNA treated HDLECS (S4 Fig) and 2) HDLEC migration in scratch assays is still significantly decreased in Zmiz1-KD compared to controls in the presence of cytosine β-D-arabinofuranoside (Fig 2K-M).

6. In Fig. S5, it is necessary to use a sample in which the expression of Zmiz1 is sufficiently decreased. At the same time, it is necessary to show that the expression of FLT4 is also downregulated by reprobing method in Western blot. Unless these are shown, the conclusion that Akt and Erk signaling is not affected cannot be drawn. Importantly, non-cropped figures should also be shown as supplementary figures.

As the reviewer advised, we tried performing western blots with VEGFR3/FLT4 antibody. However, we were unable to detect any band for VEGFR3/FLT4 on multiple tries. We used the following antibodies: AF743(R&D system) and 552857 (BD biosciences). Although we have now shown that the levels of *vegfr3/Flt4* mRNA in Zmiz1-KD studies have decreased (S5B Fig), we understand that this does not necessarily reflect the protein level or address the reviewers concern regarding this specific experiment. Therefore, after careful consideration, we decided it would be best to remove this experiment from the paper. We felt this was appropriate for 2 reasons: 1) We cannot effectively show/conclude that AKT and ERK signaling is not affected using the appropriate criteria set forth by the reviewer and 2) we believe this experiment and the corresponding results, whether loss of Zmiz1 activity affected VEGFC/VEGFR3 signaling or not, would have little impact on the overall study—ie., the absence of this experiment does not alter the main thrust of this study and its conclusions on the transcriptional role of Zmiz1. For transparency and as requested by the reviewer, we show the non-cropped western blot images used for the original manuscript, even though this experiment will now be removed.

7. In Fig. 1G and 2B, the expression of genes that are considered to be important in LECs is decreased by siRNA. To validate these data, the author should show data on the expression of these genes by qRT-PCR.

This is addressed in comment 4: we provide new qRT-PCR data showing several genes important for LEC development are downregulated when Zmiz1 is lost in HDLECs (Supporting Figure 5).

8. The author showed that when Zmiz1 expression goes down, Prox1 expression goes down. In this case, it is possible that apoptosis occurs in LECs.

Reviewer 2 brings up a good point. To address this, we performed immunofluorescent staining for the cell death marker Cleaved Caspase3 (ccasp3). We also included a positive control where we induced cell death using Staurosporine to validate the CCASP3 staining. We did not observe any cell death in either control or Zmiz1 treated cells and therefore conclude that Zmiz1-KD in LEC does not induce cell death. This data has now been included as Supporting Figure 7 and is referenced in the text.

9. Also, it is possible that tube like formation of LEC is suppressed, the author should perform the assay shown as in Fig. S4 in the article; Miyazaki et al., Cancer Sci 2014, in order to show whether the function of LECs is suppressed.

The paper referenced and titled “Expression of platelet-derived growth factor receptor β is maintained by Prox1 in lymphatic endothelial cells and is required for tumor lymphangiogenesis” by Hideki Miyazaki in 2014 showed tube like formation of LEC. The authors showed that HDLECs failed to form capillary-like structures in 3-D culture on Matrigel upon Prox1-KD. Interestingly, in our *in vivo* data, we did not observe noticeable tube formation impairment in the lymphatic vessels of the mesentery and ear in Zmiz1-KO mice (similarly addressed with Reviewer 1 and shown in new S8, S10 Figs). We feel the *in vivo* data provides evidence that tube like formation function of LECs is not affected and therefore, this in vitro tube formation assay is not critical to the study.

10. Although in Fig. 4, valve formation is decreased as a result of reduced Prox1 function, the reduced expression of FoxC2 is also expected based on the HDLEC results. The author should show the expression of Foxc2 in the valve.

In line with our transcriptomic work in HDLECs, we provided new confirmational data in S5B Fig showing that *FOXC2* mRNA is substantially reduced in HDLECs treated with the ZMIZ1 siRNA. However, we also performed western blot analysis on samples with reduced Zmiz1 expression and found no significant changes in FOXC2 protein between control and Zmiz1 siRNA (see below for the reviewer’s reference), though we did observe marked reduction in PROX1 mRNA and protein in vitro and PROX1 protein in vivo (Fig A,B).

Obviously, these results are a bit confounding. There could be various reasons for this outcome, but we can’t provide a specific explanation. Based on this data, we felt it best to place more focus on PROX1, which does show consistency between the in vitro and in vivo studies. Though we do not provide FOXC2 immunostaining of the mesenteric valves in our Zmiz1-KO mice, we still believe our in vitro data are in strong support of Zmiz1 transcriptionally regulating FOXC2 expression, though the regulation of protein expression remains a consideration for future work.

11. Since the valve formation phase is in the late stage of lymphatic differentiation, the author should analyze if the early lymphatic differentiation is affected, for example, fetal skin or whole embryo at E15.5 can be immunostained for lymphatic marker proteins.

We agree with the reviewer that the temporal aspect of lymphatic differentiation is interesting in the context of our studies. For example, are the valve defects we see in postnatal mesentery due to problems early or late in embryonic lymphatic differentiation. In the original manuscript we performed early Zmiz1 deletion at E10.5 and assessed the embryos for edema at E14.5 (no edema was found). In keeping with this same early stage as a point of comparison, we also examined the mesenteric lymphatics vessels by immunostaining for lymphatic protein markers (PROX1 and VEGFR3), as suggested by the reviewer. Intriguingly, we did not see any obvious morphological defects in lymphatic mesenteric vessels, nor did we observe downregulation of PROX1 or VEGFR3 expression, though PROX1 reduction was obvious in postnatal Zmiz1-KO mice. This may suggest temporal regulation of Prox1 expression by Zmiz1 in later lymphatic differentiation, but not during early stages of lymphatic specification and early embryonic lymphatic development. This point is now brought up in the discussion as it gives indication that Zmiz1 may have a transcriptional maintenance role for Prox1, which may explain why valve development was impacted during postnatal lymphatic differentiation. This data has been added to Supporting Figure 11, which also contains the previous embryo studies looking at edema.

Minor points: The description needs to be changed.

The following statement in the Introduction

‘Zmiz1 function is critical in diverse developmental processes, such as angiogenesis, as demonstrated by the occurrence of vascular defects and embryonic lethality upon global deletion of Zmiz1(9).’

The author should describe in more detail as this is considered an important result in vascular biology.

We have added the following sentence after to provide more details as requested by the reviewer. “Specifically, global deletion of Zmiz1 resulted in embryonic lethality at E10.5 due to vascular defects such as an underdeveloped heart valve and atrium and defective yolk sac vasculature in the Zmiz1 knockout mice.”

The author should explain what type of ECs, EC1 and EC2 are in Fig. 1B, since it is not clear what type of ECs they are.

The article, “A molecular atlas of cell types and zonation in the brain vasculature” (Vanlandewijck et al. 2018) described EC1 and EC2 as clusters of EC population and is based on transcriptional profile and clustering algorithm. Both EC1 and EC2 are denoted as endothelial cells, and are distinct from aortic, venous, or capillary ECs. This description has been added to the figure legend to provide a better description of these EC types.

Additional information for the reviewer: to classify the cells into different cell types, authors used BackSPIN algorithm and assigned endothelial cell clusters that fell outside the arteriovenous zone as EC1–EC3 and showed high expression of ribosomal protein transcripts (Rpl and Rps classes). EC1–EC3 clusters cells are distributed across all vessel types.

In Fig.S6, the author did not explain the role of Vash1 in lymphatic vessels, potential reader will not understand and the data will be meaningless. What is the role of Vash1? Also show a reference. Similarly, the role of TMEM204 in fig. 1G needs to be described.

We have added the following description on the role of Vash1 and Tmem204 in the results section corresponding to Figure 1 to provide better context of the significance of these genes to lymphatic development.

“Vash1 regulates secondary sprouting and EC proliferation and its depletion leads to defective trunk lymphatic vessel formation in the zebrafish trunk (De Oliveira *et al.*, 2021). TMEM204, also known as claudin like protein 24, is known to interact with VEGFR2 and 3 and regulates lymphatic vessel patterning and development (Saharinen *et al.*, 2010).”

In discussion section, the following was described.

‘These genes induce molecular changes that stimulate LEC migration and proliferation.’ This fact is not proven in this paper, so the author should refer to and describe the results in other articles.

We agree that this statement is not proven in our manuscript and would require further proof in this context. We were simply making the connection that these genes are downregulated in our studies and important for migration and proliferation of LEC. To this end, we added references, though we did not describe all published results, as we felt it would unnecessarily extend the discussion, which has been increased in the revision to include other points we thought were slightly more pertinent to our studies.

As to the sentence, ‘We also observed a reduction in heart valve development genes such as Dll4, Bmp2, Hey1, Snai1, and TGFβ1.’, the author should show as reference articles that demonstrated that these genes are involved in lymphatic valve formation.

The sentence would be nice if these genes were involved in both heart valve and lymphatic valve formation otherwise there is no point in listing them here.

We agree with the reviewer’s comment. Heart valve development genes such as Dll4, Bmp2, Hey1, Snai1, and TGFβ1 are downregulated in Zmiz1-KD HDLECs, but there is no data indicating a role in lymphatic valve development to our knowledge. In the discussion, we were trying to infer that Zmiz1 regulation of heart valve development genes may be an important line of study in terms of lymphatic valve development. To keep this point and align with the request of the reviewer, we have taken out the gene names and edited the sentence to the following: “We also observed a reduction in cardiac valve development genes with unknown roles in lymphatic valve development that may provide future lines of study (Fig 1I), as these genes are important for valvulogenesis and their dysregulation can lead to valve malformation or pathogenesis in the heart (E. and E., 2013; Wirrig and Yutzey, 2014)”. We hope this change is acceptable to the reviewer.

Choi, D. *et al.* (2017) ‘ORAI1 Activates Proliferation of Lymphatic Endothelial Cells in Response to Laminar Flow Through Krüppel-Like Factors 2 and 4’, *Circulation Research*, 120(9), pp. 1426–1439. doi:10.1161/CIRCRESAHA.116.309548.

E., E. and E., K. (2013) ‘Developmental Pathways in CAVD’, in *Calcific Aortic Valve Disease*. InTech. doi:10.5772/54356.

Fukasawa, K. *et al.* (2021) ‘Endothelial-specific depletion of TGF-β signaling affects lymphatic function’, *Inflammation and Regeneration*, 41(1). doi:10.1186/s41232-021-00185-4.

Itoh, F. and Watabe, T. (2022) ‘TGF-β signaling in lymphatic vascular vessel formation and maintenance’, *Frontiers in Physiology*. Frontiers Media S.A. doi:10.3389/fphys.2022.1081376.

Niimi, K. *et al.* (2021) ‘FOXO1 represses lymphatic valve formation and maintenance via PRDM1’, *Cell Reports*, 37(9). doi:10.1016/j.celrep.2021.110048.

De Oliveira, M.B. *et al.* (2021) ‘Vasohibin 1 selectively regulates secondary sprouting and lymphangiogenesis in the zebrafish trunk’, *Development (Cambridge)*, 148(4). doi:10.1242/dev.194993.

Saharinen, P. *et al.* (2010) ‘Claudin-like protein 24 interacts with the VEGFR-2 and VEGFR-3 pathways and regulates lymphatic vessel development’, *Genes and Development*, 24(9), pp. 875–880. doi:10.1101/gad.565010.

Scallan, J.P. *et al.* (2021) ‘Foxo1 deletion promotes the growth of new lymphatic valves’, *Journal of Clinical Investigation*, 131(14). doi:10.1172/JCI142341.

Sharma, M. *et al.* (2003) ‘hZimp10 is an androgen receptor co-activator and forms a complex with SUMO-1 at replication foci’, *EMBO Journal*, 22(22). doi:10.1093/emboj/cdg585.

Wirrig, E.E. and Yutzey, K.E. (2014) ‘Conserved transcriptional regulatory mechanisms in aortic valve development and disease’, *Arteriosclerosis, Thrombosis, and Vascular Biology*, 34(4), pp. 737–741. doi:10.1161/ATVBAHA.113.302071.
