# Supplementary material for: Zmiz1 is a novel regulator of lymphatic endothelial cell gene expression and function
Source: PLoS One. 2024 May 8;19(5):e0302926. doi: 10.1371/journal.pone.0302926 (PMC11078365; doi:10.1371/journal.pone.0302926)
Supplement: Attachment — Submitted filename: Response to Reviewers.docx [file pone.0302926.s016.docx]

**Reviewer #2**: The authors have experimented and responded sincerely to most of the comments. However, I believe the following two points are still critically important.

1. Not examining Sumolylation of Prox1 is not an appropriate attitude of an author, as it lacks respect for the work of past researchers who examined Sumoylation of Prox1. They did not even include a reference and to not discuss the issue. It is not natural to simply not examine the effect of Prox1 Sumolylation because this is not a difficult experiment.

With guidance from the Editor, and specifically in respect to lymphatic ECs, we performed the following assay to address Reviewer #2’s demand. We treated HDLECs with control and *Zmiz1* siRNAs (48 hours) and assessed PROX1 SUMOylation by immunoprecipitating PROX1 and then immunoblotting for both PROX1 and SUMO1. Based on the results (shown below), and as best as we can tell, we did not see any changes in PROX1 SUMOylation (performed in triplicate; one blot shown). We note that as expected and previously shown in our studies, PROX1 levels were markedly reduced in *Zmiz1* siRNA treated HDLECs compared to controls. Immunostaining for SUMO1 indicated protein detection at the same size that PROX1 was immuno-detected in both control and *Zmiz1* siRNA HDLECs. This might suggest detection of a SUMOylated form of PROX1, though no obvious changes in typical PROX1 size or additional larger bands were observed as in the Pan et al., studies (denaturing and non-denaturing conditions showed similar results in our experiments). However, we note that the Pan et al., studies used non-lymphatic cells and the one vascular cell line they used was a fusion of HUVEC to a human lung cancer cell. Thus, it is unclear if PROX1 SUMOylation takes place in lymphatic cells, though it clearly does in other cell types.

Densitometric measurements of PROX1/SUMO1 ratios in control and *Zmiz1* siRNA treated HDLECs showed no statistical differences in expression levels. Thus, based on the lack of an additional, larger size PROX1 band and no changes in SUMO1 expression levels, our experiments indicate loss of Zmiz1 did not have an effect on PROX1 SUMOylation, at least via SUMO1. Since the reviewer was adamant about performing PROX1 SUMOylation experiments, we have included these findings as S7 Fig with additional text being incorporated into the results section and S7 Fig legend. However, we note that we are not confident that these results show or do not show Zmiz1 mediated PROX1 SUMOylation in lymphatic ECs.

We felt it most important and relevant to conduct the experiments in HDLECs, as opposed to non-lymphatic EC types, which were used in the reference provided by the reviewer. However, to further assess possible SUMOylation of PROX1 by ZMIZ1, we performed an additional experiment in human 293T cells, similar to the original suggestion of the reviewer and similar to experiments in the reference provided by the reviewer. Epitope tagged versions of ZMIZ1 (Zmiz1-FLAG; cloned in our lab) and SUMO1-HA (Addgene, 21154) were transfected independently and together in 293T cells for 48 hours. Proteins were then subjected to western blot analysis (shown below). Immunoblotting for HA in 293T cells transfected with SUMO1-HA alone showed a strong band at approximately 95 kDa, which also coincided with the size for PROX1 seen in all samples. This result was similar to the experiment above and may indicate that PROX1 was SUMOylated by SUMO1. On the other hand, staining for PROX1 itself only resulted in a single band at ~95 kDa and not a second larger band that might be expected if some of PROX1 was SUMOylated. Zmiz1-FLAG transfected 293T cells displayed a band at the expected 115 kDa size (arrow), though this band was moderate in signal strength and directly above a non-specific band. Importantly, transfection of constructs for both epitope-tagged proteins revealed no obvious changes in size of PROX1 or additional larger PROX1 bands as compared to the control or single transfected lanes. Furthermore, HA immunostaining showed the same size band (~95 kDa) as in the SUMO1-HA individually transfected cells, while ZMIZ1 was also detected. However, we noted that expression of these proteins, including PROX1 appeared reduced proportionally; we don’t have an explanation for this aspect of the experiment. Ultimately, these results may indicate PROX1 SUMOylation was unaltered in the presence of excess ZMIZ1, though admittedly, it’s difficult to interpret. For this reason, we have shown the images of the experiment to the reviewer below but have not included it into the paper. We hope this demonstrates to the reviewer that our experiments were conducted in good faith but because of the results we were reluctant to include them into the final paper. Moreover, and as indicated with the previous experiment, we are also hesitant to include the above experiment (PROX1 IP in HDLECs) in the paper, but ultimately did because of the reviewer’s request. Though it is our opinion, that these experiments: a) were not as easy to conduct/interpret as was indicated by the reviewer, b) may not sufficiently address the reviewer’s request c) still do not alter the *main thrust of the paper that Zmiz1 transcriptionally regulates lymphatic gene expression*, *including Prox1* and d) might be better left off entirely from the manuscript, as we don’t feel they are conclusive, though we have tried diligently to assess SUMOylation of Prox1 via Zmiz1 and further investigation would take much longer than we feel is warranted considering our efforts into the manuscript studies.

2. Even though there are indivisual siRNAs, the authors did not examine the off-target effect of siRNAs, it is not appropriate response because it may downgrade the scientific quality of the data.

As noted previously, Zmiz1 siRNA were obtained as a SMARTpool ON-TARGETplus siRNA from Horizon Discovery, which is a mixture of 4 siRNAs provided as a single reagent—providing advantages in both potency and specificity. To address the reviewer’s concern about potential off-target effects, we performed siRNA transfections in HDLECs with 2 of the 4 Zmiz1 siRNAs individually and compared them to control siRNA treated cells—2 independent Zmiz1 siRNAs were tested based on the guidance of the Editor. After 48 hours of treatment, RNAs were isolated and qPCR analysis was conducted to assess mRNA levels of *ZMIZ1, PROX1, EFNB2, FLT, COUPTFII*, and *FOXC2*. All mRNAs assessed were substantially reduced, similar to previous results utilizing the Zmiz1 SMARTpool siRNAs. Thus, the results indicate high specificity of the Zmiz1 siRNAs and subsequently suggest limited off-target effects. The results of these experiments are noted in the methods section and the data is presented as an additional part of S5 Fig. We believe these experiments address the reviewer’s concern over potential Zmiz1 siRNA off-target effects.

Minor point:
Listing the references in No. 54 and 55 is not balanced in context. The original articles, not the review article should be added as reference articles

This was an error on our part. We thank the reviewer for catching this and have subsequently rebalanced the references and used the original articles in citation #56 and #57.
