# Supplementary material for: Zmiz1 is a novel regulator of lymphatic endothelial cell gene expression and function
Source: PLoS One. 2024 May 8;19(5):e0302926. doi: 10.1371/journal.pone.0302926 (PMC11078365; doi:10.1371/journal.pone.0302926)
Supplement: Attachment — Submitted filename: Response to reviewer #2_Revision 3.docx [file pone.0302926.s017.docx]

**Reviewer #2**: Since a review is a communication between the author and the reviewer,
It sounds very rude to say 'Adamant'. Experiments or modifications requested by the reviewer could be rejected by the authors if they can be scientifically justified or stated.
The author's scientific communication skills are disappointing, but I admit the new data presented by authors is the results of their efforts. This revision could be acceptable if the following points can be addressed.

Pan's group has shown by immunoprecipitation experiments that SUMOylation of Prox1 occurs using LEC (Fig. 1C). Therefore, the author is wrong in assuming that we do not know whether SUMOylation of Prox1 occurs in LECs. This is disappointing because it shows that the author is not capable of carefully reading the prior report.

Also, and more importantly, that the Prox1 band is shown at 95 kDa,
It is more natural to assume that Prox1 is originally estimated to be around 83-85 KDa and that most Prox1 is SUMOylated. The author misinterpreted the experimental facts by missing them. At the same time, Pan's group may have mistakenly assumed that the 95 kDa protein is deSUMOylated form of Prox1.

Therefore, most of Prox1 is considered to be SUMOylated at this point.
Then the experiment of overexpression of SUMO and Zmiz1 using 293T is not very meaningful.

Supplementary figure 7 does not show that the cell extracts used for IP had the same amount of protein, and the quantification method was wrong.
The data should originally show that the same protein amounts were used in the control siRNA and Zmiz1 siRNA samples.
More precisely, the graph in fig. S7B should be shown after showing the amount of internal control protein is equally loaded. Otherwise, it is meaningless.

This is a basic rule when performing immunoprecipitaion assay. Therefore, this experiment needs to be redone. However, since it is understood that to investigate SUMOylation of Prox1 in depth, experiments such as SUMO1 knockdown will be necessary, I do not demand the authors further it in this revision.

**Considering the above, Supplementary Figure 7 and the related text can be removed.**

We have removed S7 Fig and the related text, and subsequently reordered the supplementary figures remaining in the manuscript.

However, please explain the discrepancy since Prox1 is shown at 85 kDa in Fig. 4 and 95 kDa in figure S7. This is a problem of the reliability of the authors’ data and ability to interpret the data.

Please see raw images of Fig 4 and previously S7 Fig below. In both cases, you will see that the PROX1 bands appear to be in the same location in the blots (between the 100 kDa and 75 kDa molecular weight markers; dotted lines were added to help better see this as well). Therefore, we don’t believe there are any discrepancies between the experiments—we believe we are picking up the same sized PROX1 band. We indicated the PROX1 band at 83 kDa in Fig 4 because the antibody used from Abcam (ab199359) predicts a size of 83 kDa via western blot and is how they label it, notwithstanding a potential SUMOylated form of PROX1. Of course, it is difficult to know the exact size based on the difference between the molecular marker sizes. In S7 Fig, we noted the PROX1 band at ~95 kDa primarily based on 2 factors, though this may or may not have been correct. Pan’s group indicated PROX1 band sizes at 95 kDA and 110 kDA when SUMOylated. It was clear that our band was not 110 kDa. Since immunoblotting for SUMO1 and PROX1 in the IP experiments suggested detection of a SUMOylated form in between 100 – 75 kDa, we thought it was most likely the 95 kDA version of PROX1 and thus used this size description. However, it is difficult to know the exact size, as there might not be an easily noticeable difference between 83 and 95 kDa. In looking at both blots, there doesn’t appear to be any noticeable differences in size and thus we don’t believe there are discrepancies, but rather our error in how we perceived the sizes. We may be picking up SUMOylated PROX1 in Fig 4, but since we have removed S7 Fig and because it is difficult to compare and know the exact size of our PROX1 bands with Pan’s group (plus SUMOylated versus non-SUMOylated bands), we decided to take out any specific reference to size and instead show the 100 and 75 kDa molecular sizes as reference (see updated Fig 4a below).

Fig 4a
